# FeNxC Based Catalysts Prepared by the Calcination of Iron-Ethylenediamine@Polyaniline as the Cathode-Catalyst of Proton Exchange Membrane Fuel Cell

**DOI:** 10.3390/polym11081368

**Published:** 2019-08-19

**Authors:** Yen-Zen Wang, Wen-Yao Huang, Tar-Hwa Hsieh, Li-Cheng Jheng, Ko-Shan Ho, Sin-Wei Huang, Liang Chao

**Affiliations:** 1 Department of Chemical and Materials Engineering, National Yun-Lin University of Science and Technology, 640 Yun-Lin, Taiwan; 2 Department of Photonics, National Sun Yat-sen University, 70 Lienhai Rd., Kaohsiung 80424, Taiwan; 3 Department of Chemical and Materials Engineering, National Kaohsiung University of Science and Technology, 415, Chien-Kuo Road, Kaohsiung 80782, Taiwan; 4 Center for General Education (Math), Taipei City University of Science and Technology, 2 Xueyuan Rd., Beitou, Taipei 112, Taiwan

**Keywords:** FeNxC catalyst, ethylene diamine, polyaniline, two-stage calcination

## Abstract

Calcinated tris(ethylenediamine)iron(III) chloride was used as a non-precious metal catalyst (NPMCs) for a proton exchanged membrane fuel cell (PEMFC) under the protection of polyaniline (PANI), which behaves as both nitrogen source and carbon supporter. The optimal ratio of FeCl_3_/EDA was found to be close to 1/3 under the consideration of the electrocatalytic performance, such as better oxygen reduction reaction (ORR) and higher power density. Two-stage calcination, one at 900 °C in N_2_ and the other at 800 °C in mixed gases of N_2_ and NH_3_, result in an FeNxC catalyst (FeNC-900-800-A) with pretty high specific surface area of 1098 m^2^·g^−1^ covered with both micro- and mesopores. The ORR active sites focused mainly on Fe–Nx bonding made of various pyridinic, pyrrolic, and graphitic N-s after calcination. The max. power density reaches 140 mW·cm^−2^ for FeNC-900-800-A, which is superior to other FeNxC catalysts, experiencing only one-stage calcination in N_2_. The FeNxC demonstrates only 10 mV half-wave-voltage (HWV) loss at 1600 rpm after 1000 redox cycles, as compared to be 27 mV for commercial Pt/C catalyst in the durability test.

## 1. Introduction

The expensive precious metals used as oxygen reduction reaction (ORR) catalysts restrain the mass-production of proton exchange membrane fuel cells (PEMFCs). Non-precious metal catalysts are suitable candidates to substitute Pt catalysts. The aim of non-precious metal catalysts (NPMCs) is to substitute high priced Pt-based catalysts. NPMCs require not only high ORR catalytic activity but also inexpensive components. The developing carbon based inexpensive non-precious metal ORR catalyst has turned out to be one of the most interesting topics in the area of fuel cells.

The chemical reaction of a fuel cell is based on the redox reaction between hydrogen and oxygen in the anode and cathode, respectively. However, ORR at the cathode is much slower than that of the hydrogen reduction reaction (HOR) at the anode, which needs more precious Pt-catalyst to pick up the ORR rate to fulfill the redox cycle to produce power. In other words, the ORR has turned out to be the neck-intermediate reaction during the redox reaction. And the main factor to cause the delay comes from the intermediate product of hydrogen peroxide (H_2_O_2_), leading to a delay and more Pt-catalyst consumption.

Recently, a new type of non-precious catalyst based on the coordinated metal–Nitrogen- carbon (MNC) was developed, which proves it can provide a steady improvement of ORR and become a strong candidate to replace the expensive Pt-catalyst. Besides, the MNC catalysts are less sensitive to the presence of chloric ions, which are usually found in the proton exchanged membrane fuel cell (PEMFC) [1,2,3].

The first MNC catalyst was prepared when cobalt was used to coordinate with a large cycled cobalt phthalocyanine (Co-P-C) by Jasinski in 1965 [4]. Bagotzky modified the catalyst (Co-porphyrin, CoTMPP) the next year and again treated at high temperature, which did not improve the efficiency of the catalyst, but the ORR was greatly improved [5]. Therefore, people understand calcination higher than 800 °C is necessary to prepare an MNC type of catalyst. N-containing polyacrolonitrile (PAN) was used to prepare MNC as an N-source in 1989 by Yeager. And both FeNxC and CoNC demonstrated comparable catalyzing efficiency and ORR with CoTMPP after pyrolysis at 800 °C [6]. The coordinated structure of FeNxC after calcination was confirmed later by more sophisticated characterization later. And more N-sources like cyanamide [7,8], aliphatic [9], and aromatic [10,11] amino compounds were applied to coordinate with Fe for the calcination process that followed.

Some carbide-derived carbon (CDC) [12] were modified to have all kinds of pore size distributions in the micro- or mesoporous areas of FeNxC, and the numbers of active sites can be defined. They find some important key points that possibly derived from the existence of the second type of atoms from the original carbide compound. Mamtani [13] prepare an FeNxC at temperature higher than 950 in the argon and ammonia atmosphere. The catalyst demonstrated significant improvement in ORR activity after acid washing away magnetic compounds of FeO, Fe_2_O_3_. Jiang [14] found the activity of Fe–Nx can be boosted with the formation of Fe/Fe_3_C nanocrystals in 2016. Sulfur proved to be able to improve the catalytic performance of FeNxC by Luo [15]. Some amines were used to form a complex with Fe ions and calcinated into FeNxC catalysts. Li [16] used ethylene diamine (EDA) in the preparation of FeNxC as the catalyst for an alkaline Fuel cell (AFC). N-rich pentaethylenehexamines were used to complex with FeCl_3_ on the Vulcan XC-72 (carbon black) surface to improve the tolerance of methanol crossover during ORR in a direct methanol fuel cell (DMFC) [17]. EDA were used as an N-precursor when an Fe-Co−N*_x_* compound was loaded onto carbon black with nanoporous sizes [18]. Ultra-high surface area of the graphitic FeNxC nanospheres with only one iron atom were designed to improve ORR [19,20,21,22]. Recently, attention has been paid to create more active sites of Fe–N by dispersing Fe as atoms. Their structural and electronic structures related to the carbon matrix have also been studied [23,24,25,26,27,28,29,30].

In this study, to save the tedious approaches for synthesizing large cycled iron phthalocyanine metal-organic-complex, we applied both aliphatic and aromatic amines as the N- and C-sources, respectively, whose amino groups can chelate with Fe ions before calcination. Aliphatic amines like EDA can easily form complexes with Fe ions due to its dexterous light weight to become an insoluble salt in the aqueous system, which guarantees the coordination of Fe ions with EDA molecules. However, the Fe–N complex does not easily maintain at high temperature calcination due to the easy thermal degradation of low molecular weight EDA. During the conversion from chelated Fe^3+^…N into covalent bond (Fe–N), the EDA itself might degrade and de-complex from Fe ions before it forms a covalent bond with Fe (Fe–Nx) during calcination, which also effectively decreases the percentage of N- in the final product. To protect the EDA from thermal degradation and to provide more N-sources, the salts made of chelated Fe^3+^…N were mixed with aniline monomers before polymerization. Therefore, iron compounds can be surrounded and protected by polyaniline (PANI) after polymerization before being subjected to calcination, and most of the protected Fe-EDA are able to safely convert to covalent network of FeNxC which can be used to substitute Pt-catalyst as the cathode-catalyst in the PEMFC system. The ORR efficiency and power density of the single cell will be measured.

## 2. Experimental

### 2.1. Materials

Aniline (Tokyo Kasei Kogyo Co., Ltd., Japan), hydrochloric acid (Scharlau, Barcelona, Spain), ammonium hydroxide (J.T.Baker, USA), ammonium persulfate (J.T.Baker, USA), Iron(III) chloride hexahydrate (FeCl_3_·6H_2_O, J.T. Baker, USA), ethylenediamine (J.T. Baker, USA).

### 2.2. Preparation of FeNxC Catalyst

1 g (0.0037 mol) of Iron(III) chloride hexahydrate was mixed with 1 M HCl (aq.) solution and stirred to become uniform. 1 mL (0.66 g:0.011 mol) of EDA was introduced into the mixture (the molar ratio Fe/EDA = 1/3) and stirred again, followed by the addition of 2 g aniline monomers. 4.9 g of ammonium persulfate (APS) dissolved in 1 M HCl (aq.) was slowly added into the previous mixture to start the polymerization until the mixed solution became dark green (about 1.5 h). After the polymerization, the solvents of the mixture were removed by vacuum oven at 80 °C and the precursor of the FeNxC catalyst was obtained. The precursor was heated to 700 °C (800, 900, 950 °C) at 10 °C min^−1^ and stayed for 1 h in the argon atmosphere, then cooled to room temperature (RT). These samples were named FeNxC-700 (-800, -900, -950). The impurities and magnetic materials of the obtained FeNxCs were removed by washing in 1 M H_2_SO_4_ (aq.) at 80 °C for 3 h, followed by drying in a vacuum oven at 60 °C. These sample were named FeNxC-700-A (-800-A, -900-A, -950-A). FeNxC-900-A was further calcinated to 800 °C in N_2_ and NH_3_ atmospheres again at 10 °C min^−1^ (named FeNxC-900-800) and washed again in 1 M H_2_SO_4_ (aq.) at 80 °C for 3 h, followed by drying in a vacuum oven at 60 °C. The sample was named FeNxC-900-800-A.

### 2.3. FTIR

The main functional groups of neat PANI and various FeNxCs prepared with different ratios of FeCl_3_/EDA were assigned from FTIR spectra which were recorded on an IFS3000 v/s FTIR spectrometer (Bruker, Ettlingen, Germany) at room temperature with a resolution of 4 cm^−1^ and 16 scanning steps.

### 2.4. X-ray Photoelectron Spectroscopy (XPS)

The different binding energy spectra of N1s of various FeNxCs were used to characterize the percentage of pyridinic, pyrrolic, graphitic Ns, etc. after calcination by an XPS instrument of Fison (VG)-Escalab 210 (Fison, Glasgow, UK) using Al Ka X-ray source at 1486.6 eV. The pressure in the chamber was maintained under 10^−6^ Pa or lower during the measurement. A tablet sample was prepared by a stapler. The binding energies of the N1s around 400 eV were recorded.

### 2.5. WXRD (Wide angle X-ray Diffraction: Powder X-ray Diffraction)

A copper target (Cu-Kα) Rigaku x-ray source (Rigaku, Tokyo, Japan) with a wavelength of 1.5402 Å was used for x-ray diffraction. The scanning angle (2θ) started from 10° to 90° with a voltage of 40 kV and a current of 30 mA, operated at 1° min^−1^.

### 2.6. Energy Dispersive X-ray Spectra (EDx)

The EDx spectra of various FeNxCs were obtained from an XL-40EFG by Philips (Amsterdam, Netherlands). The samples were coated with gold before measurement.

### 2.7. Scanning Electronic Microscopy (SEM)

The sizes and morphologies of FeNxCs were characterized by SEM (field emission gun scanning electron microscope, AURIGAFE, Zeiss, Germany).

### 2.8. Surface Area and Pore Size Measurement (BET Method)

Nitrogen adsorption–desorption isotherms (type IV) are obtained from an Autosorb IQ gas sorption analyzer (Manufacturer, city, country) (Quantachrome) at 25 °C. Samples are degassed at a temperature higher than 100 °C overnight. The surface area was calculated according to the BET equation when a linear BET plot with a positive C value was in the relative pressure range. Pore size distribution was determined by the Quenched Solid Density Functional Theory (QSDFT) methods based on a model of slit/cylinder pores. The total pore volumes were determined at P/P_0_ = 0.95.

### 2.9. Electrochemical Characterization

#### 2.9.1. Current–Potential Polarization-Linear Sweep Voltammetry (LSV)

The performance of the electrocatalyst support was tested with a three-electrode system. The round working electrode with an area of 1.5 cm^2^ was prepared as follows: Ag/AgCl and carbon graphite were used as the reference and relative electrode, respectively. The electrochemical test was carried out in a potentiostat/galvanostat (Autolab-PGSTAT 30 Eco Chemie, Artisan Technology Group, IL, USA) in 0.5 M H_2_SO_4_ solution and C-V curves were obtained with scanning potential from −0.2 to 1.0 V at a sweeping rate of 50 mV·s^−1^. The catalyst ink was prepared by mixing 3mg support powder in isopropanol and stirred until it became uniform. Subsequently, 5% Nafion solution was added into the mixture as binder and the mixture was ultra-sonicated for 1 h and the obtained ink was uniformly spray-coated on the carbon paper for C-V test.

The current-potential polarization curves obtained from LSV of the various FeNxCs were measured using a rotating-disk electrode (RDE) operating at 900, 1200, 1600, 2500, and 3600 rpm in O_2_-saturated 0.5 M H_2_SO_4_, respectively. The reduced current densities of various FeNxCs, which were recorded at 1600 rpm within the measured voltage range (−1.2 ~ 0.5 V) were chosen to compare with each other.

#### 2.9.2. MEA Preparation

A Nafion^®^ 212 sheet purchased from Ion Power Inc., New Castle, DE, USA was used as the proton exchange membranes. In order to remove the surface organic impurities and to convert the membranes into protonated (H^+^) form, the Nafion-212 (4 × 4 cm^2^) membrane was treated at 70 °C in 5 wt.% H_2_O_2_ aqueous solution for 1 h, followed by submerging in 1 M H_2_SO_4_ solution for 1 h and subsequently the treated membranes were dipped in distilled water for 15 min and stored in de-ionized water. The catalyst inks were prepared by mixing 20 mg of FeNxC powders in isopropanol and mechanically stirred until it became uniform before 5% Nafion solution was added. Eventually, the catalyst mixture was ultra-sonicated for 1h, followed by coating on both sides of the treated Nafion sheet dropwise as anode and cathode electrodes (2 × 2 cm^2^), respectively, and hot-pressed at 140 °C with a pressure force of 70 kg cm^−2^ for 5min to obtain the MEA.

#### 2.9.3. Single-Cell Performance Testing

The MEA was installed in a fuel cell test station for testing using the single-cell test equipment (model FCED-P50; Asia Pacific Fuel Cell Technologies, Ltd., Miaoli, Taiwan). The active cell area was 2 × 2 cm^2^. The temperatures of anode, cell and cathode and humidifying gas (90% RH) were all maintained at around 70 °C. The flow rates of anode input H_2_ and the cathode input O_2_ fuels were set at 200 and 100 mL·min^−1^, respectively, based on stoichiometry. To test the electrochemical performance of FeNxC catalyst in the individual MEAs, both C-V and output powers were measured.

## 3. Results and Discussion

### 3.1. FTIR Spectra

**Scheme 1:**
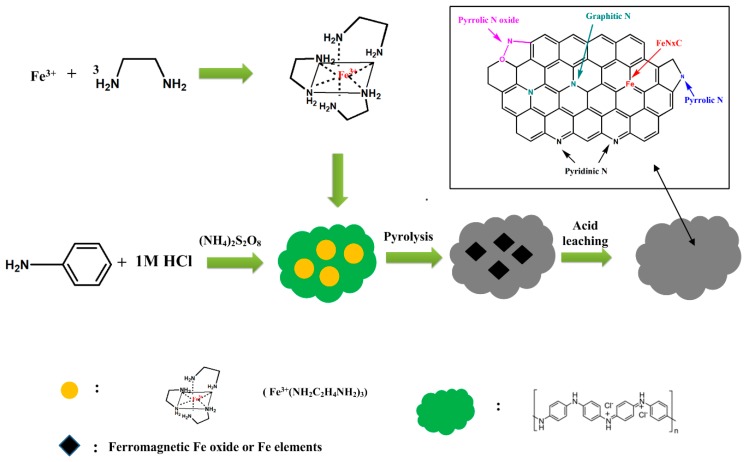
Preparation diagram of FeNxC catalyst.

Regularly, Fe^3+^ are coordinated with water in the form of Fe^3+^(H_2_O)_6_(FeCl_3_.6H_2_O) and the coming of EDAs was able to replace the water molecules. Significant green precipitation was seen when the molar ratio of FeCl_3_/EDA is close to 1/3, whose structure is thought to be Fe^3+^(NH_2_C_2_H_4_NH_2_)_3_. When more EDA was added, no further precipitation can be seen. It is believed that an insoluble salt of tri(ethylenediamine)iron(III) chloride was created as the three EDA ligands occupied the six coordination positions of ferric ion, resulting in the formation of octahedral crystal demonstrated in Scheme 1.

**Figure 1:**
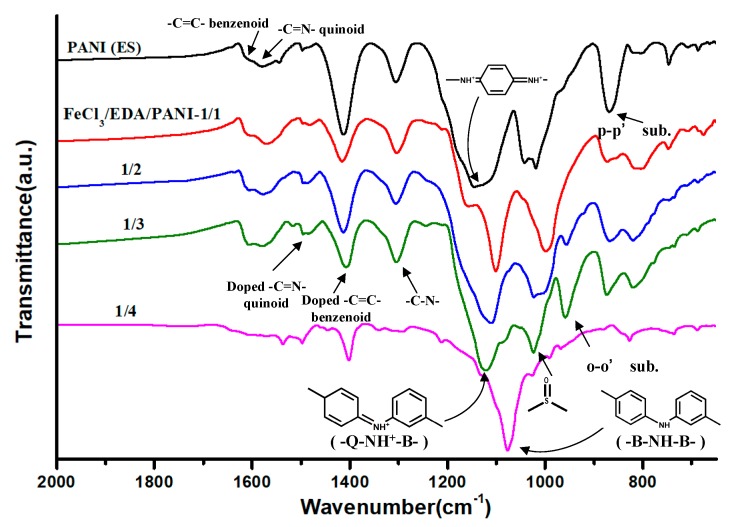
IR spectra of FeNxCs prepared with various FeCl_3_/EDA ratios.

The Fe-EDA complex was prepared with different FeCl_3_/EDA ratios before covering by PANI, which were characterized by IR-spectra and illustrated in Figure 1. Major functional groups (doped and undoped states of quinoid, benzenoid, –C–N– groups) of PANI are assigned directly as seen in Figure 1, indicating PANI (ES) was successfully obtained. However, when the FeCl_3_/EDA ratio was over 1/3 (1/4), the spectrum demonstrated more undoped –B–NH–B– than doped –Q–NH^+^–B– groups (Q: Quinoid; B: Benzenoid) due to the neutralization effect of the surplus EDA which behaves as a base chemical and can induce the dedoping of the –Q–NH^+^–B– group. The sulfonic groups assigned at 1020 cm^−1^ were derived from the persulfate initiator, which is converted to sulfuric group after the initial reaction.

A blank test without the protection of PANI produced a high magnetic FeNxC catalyst after calcinating at 900 °C. Therefore, even under the protection of PANI, the calcinated FeCl_3_/EDA@PANI with FeCl_3_/EDA ratio equal to or higher than 1/4 also still demonstrated a magnetic attraction property due to the strong reducing force of the excess EDA, which made the dispersion in the solvent very difficult during preparation of a slurry ink for the MEA. Although the magnetic compounds of the calcinated FeNxC products could be easily removed by acid-leaching when the FeCl_3_/EDA ratio was below 1/4, it could only be partly removed at a higher ratio than 1/3 (1/4). In other words, a higher FeCl_3_/EDA ratio than 1/3 would lead to a magnetic FeNxC catalyst during calcination and could not be totally removed by acid-leaching.

### 3.2. XPS

**Scheme 2:**
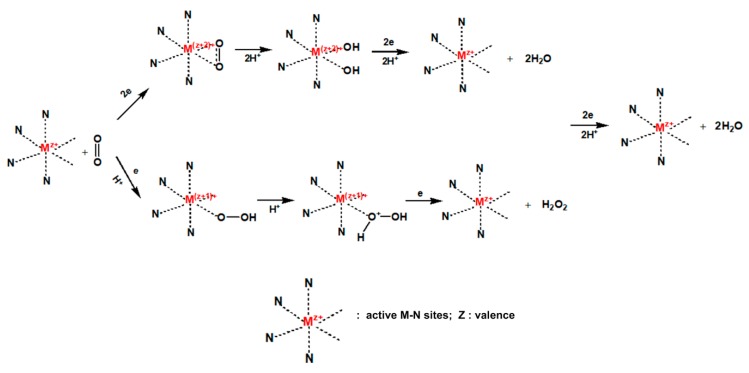
Electrocatalytic mechanism of FeNxCs.

The active sites of FeNxC can absorb O_2_ gas and form a peroxide structure which would dissociate in the presence of protons during reduction (Scheme 2). The formed peroxide would dissociate in two approaches [31,32], one becoming diol and following the four-e route, directly converting into water. The other went on consecutive reduction with two electrons involved in the following stage with H_2_O_2_ as the intermediate product (Scheme 2). The catalytic mechanism followed the traditional six-coordination catalytically reaction.

Theoretically, the O_2_ gas with two lone pairs could be absorbed by the active sites of FeNxC through the coordination, or trapped in the porous holes with various N– related bonds whose lone paired electrons can improve the O_2_ absorbing capability of FeNxC and behave as active sites like transitional metals (Fe). The active –N can be pyrrolic –N, graphitic –N (Scheme 1), but not pyridinic –N [33,34,35,36,37,38], whose lone-paired electrons were easily covered by the acidic proton through acid-base neutralization (–N: + H^+^ => –NH^+^). The first calcination at high temperatures in the N_2_ atmosphere can create various –N containing covalent bonds, behaving as active sites and the second calcination in the mixed gases of N_2_ and NH_3_ would engrave on the FeNxC surfaces with lots of micro- or mesopores, resulting in a drastically increasing surface area and exposing more active –N and –Fe sites to the O_2_ gas. Therefore, FeNxC-900-800, which experiences two stages of calcination in different atmospheres, gives the highest LSV current and max. power density, which will be discussed.

**Scheme 3:**
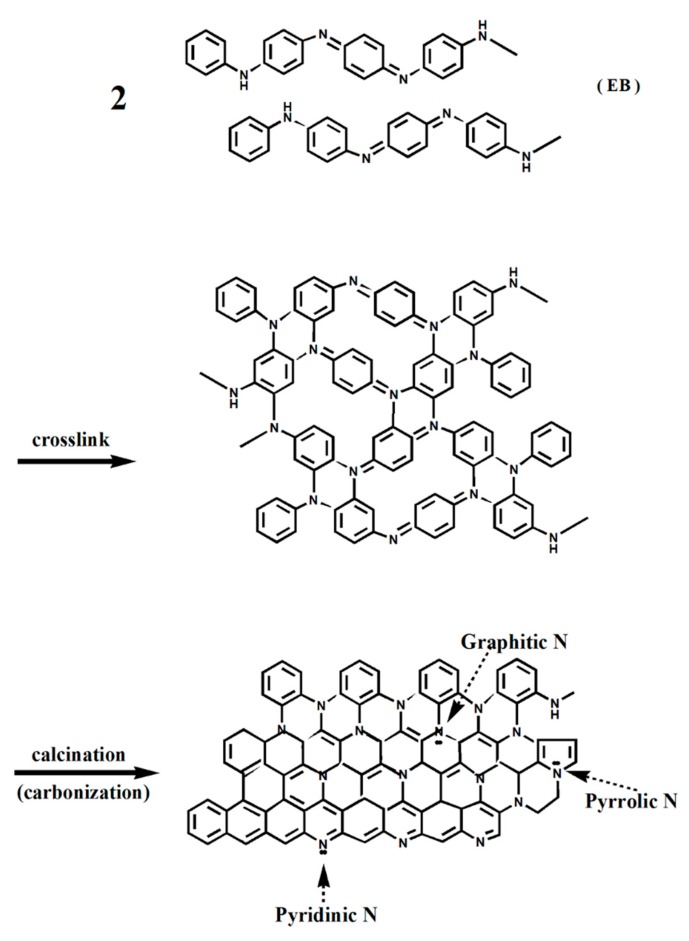
Schematic diagram of the formation of PANI networks through calcination.

**Scheme 4:**
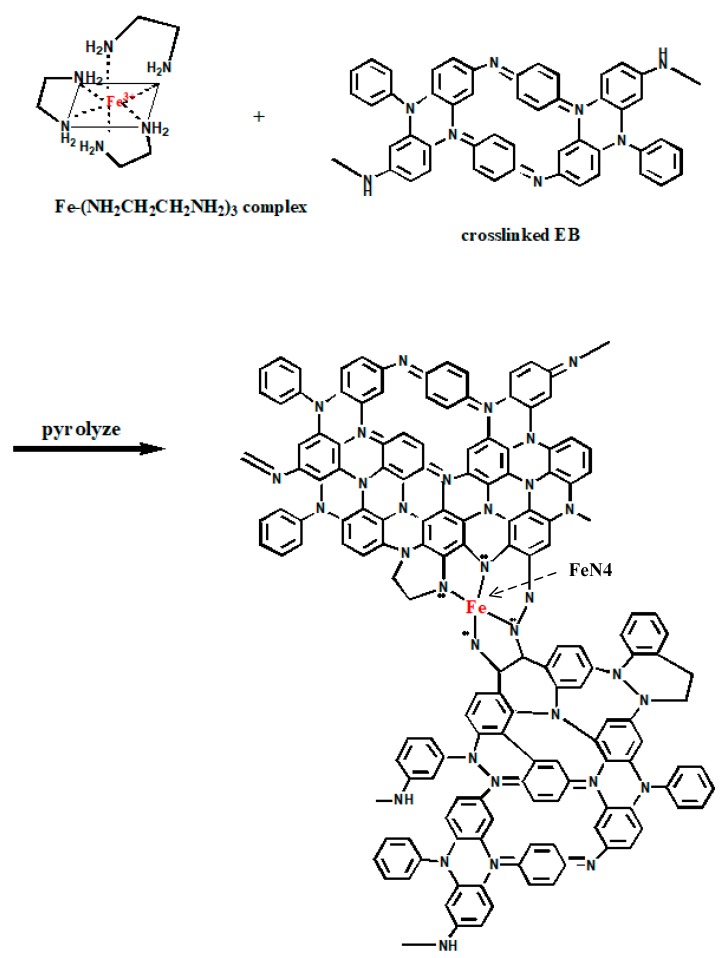
schematic diagram of formation Fe–Nx bonding between Fe and PANI.

For PANI itself, it would crosslink into ladder-like polymers with each other in the initial stage of thermal heating and higher temperature pyrolysis allows the carbonation between the ladder-like polymers, which would form networks of graphitic –N, pyridinic –N, and pyrrolic –N (Scheme 3). Both pyridinic and pyrrolic –Ns are on the edges of calcinated PANI while graphitic –N are located inside. The conjugated, carbonized structure of pyrolyzed EB can act as a conducting medium as well, allowing the flowing in of electrons from the anode. It saves the trouble of adding XC-72 in the preparation of the cathode ink. Besides, the tri(ethylenediamine)iron(III) complexes can successfully convert into a covalent network under the protection of enclosed PANI during calcination and Fe would capture more –N, becoming the active Fe–Nx centers (Scheme 4).

**Figure 2:**
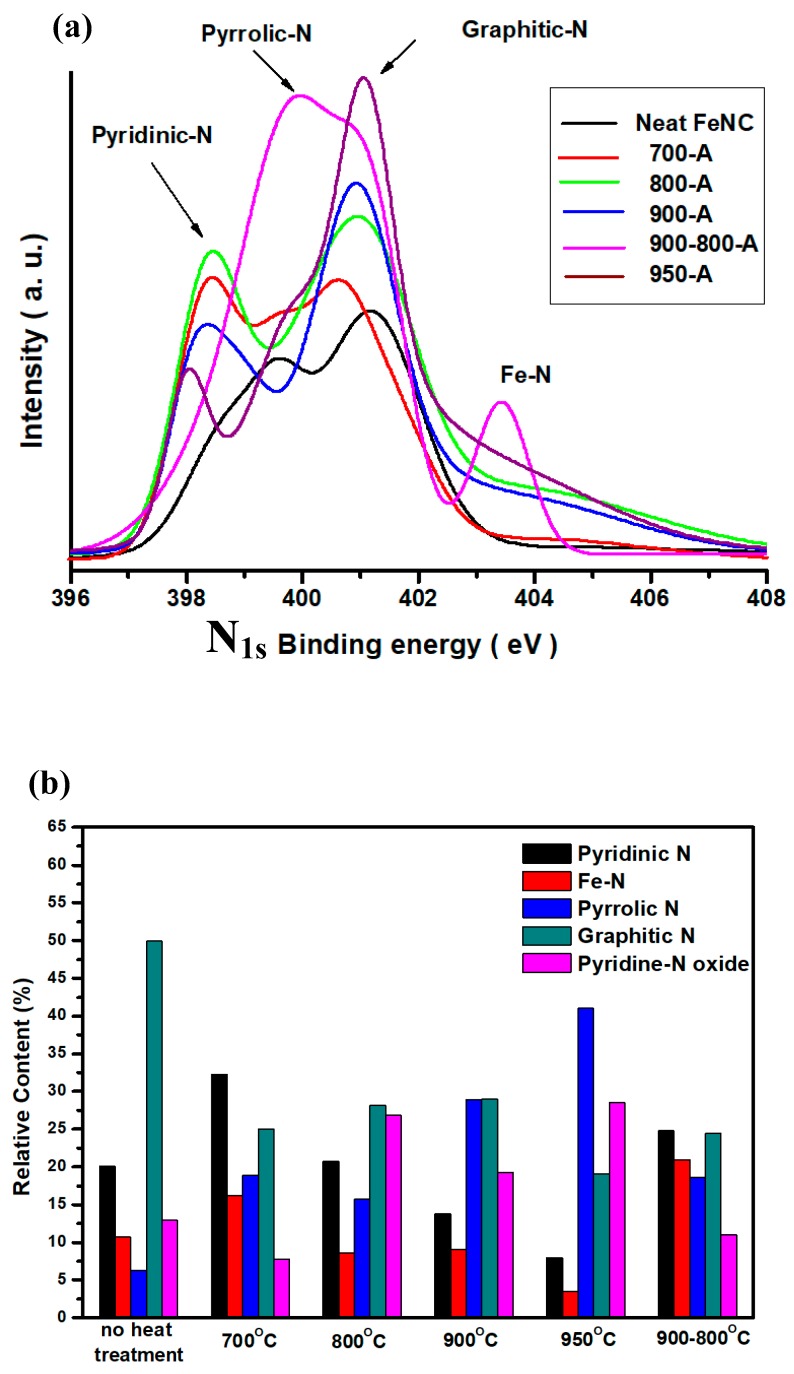
(**a**) XPS of FeNxC prepared at different temperatures, (**b**) relative compositions of each type of –Ns.

The N1s XPS spectra of FeNxCs treated at high temperatures after acid-leaching are stacked together and demonstrated in Figure 2a. The compositions of each type of –Ns are illustrated in Figure 2b. The neat and FeNxC-900-800-A demonstrate less pyridinic –Ns. More pyridinic –Ns are found for FeNxCs experiencing only one stage of calcination in N_2_ atmosphere according to Figure 2. Briefly, some of the pyridinic –Ns were converted to pyrrolic –Ns at the second calcination in the presence of NH_3_ and N_2_. Two features can be seen for the N1s XPS spectrum of FeNxC-900-800-A in Figure 2b. One is the competing high concentration of pyrrolic and graphitic –Ns and the other is that it owns the significant Fe–N covalent-bonding. High power density of the single cell prepared with a catalyst of FeNxC-900-800-A containing more graphitic, pyrrolic –Ns, and active Fe–N bonds will be discussed.

### 3.3. XRD

**Figure 3:**
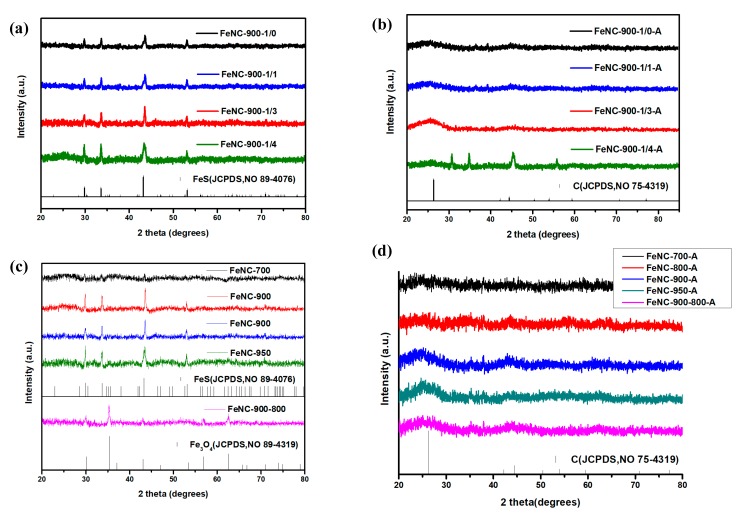
X-ray diffraction patterns of various FeNxCs.

The optimal ratio of tri(ethylenediamine)iron(III)@PANI is also checked with XRD patterns which demonstrate a significant crystalline pattern of FeS after calcination at 900 °C for all ratios in Figure 3a. However, the FeS crystalline can be removed by leaching with acid when the ratio is between 1/1 and 1/3 as shown in Figure 3b. For a sample prepared with tri(ethylenediamine)iron(III)@PANI equal to 1/4, the X-ray diffraction pattern reveals the dominating FeS crystalline structure. The initiation of aniline monomers can convert the peroxysulfate into sulfate ions and the formed FeSO_4_ can easily be converted into FeS with high temperature calcination.

XRD patterns are also used to characterize the optimal calcination (preparing) temperature of the FeNxCs. The XRD spectra and standard patterns of each specific crystalline compound are illustrated in Figure 3c,d to demonstrate the crystalline pattern of FeNxCs after treating at various temperatures and acid-leaching. All FeNxCs which experience only one-stage calcination demonstrated a high concentration of FeS crystals (Figure 3c). Only after second stage calcination at 800 °C (FeNxC-900-800) in mixed gases (N_2_ + NH_3_) the FeS can be replaced with Fe_3_O_4_ (Figure 3c). However, the high magnetic property of Fe_3_O_4_ makes further characterization by SEM and TEM impossible. Besides, strong attractive magnetic forces make the dispersion of FeNxC-900-800 in the isopropanol solvent very difficult, leading to no MEA that can be successfully prepared. Therefore, the acid-leaching procedure is necessary and can remove most of the FeS or Fe_3_O_4_ crystallines for all FeNxCs except that one prepared with FeCl_3_/EDA equal to 1/4 according to Figure 3d. After removing the FeS or Fe_3_O_4_ compounds by acid-leaching, the spaces, once occupied by them, become many nanopores and mesopores on the surfaces of FeNxCs, which can expose more active sites of Fe–N or create more empty, tiny spaces to accommodate more oxygen gas for better electrocatalytic properties.

### 3.4. EDx

**Figure 4:**
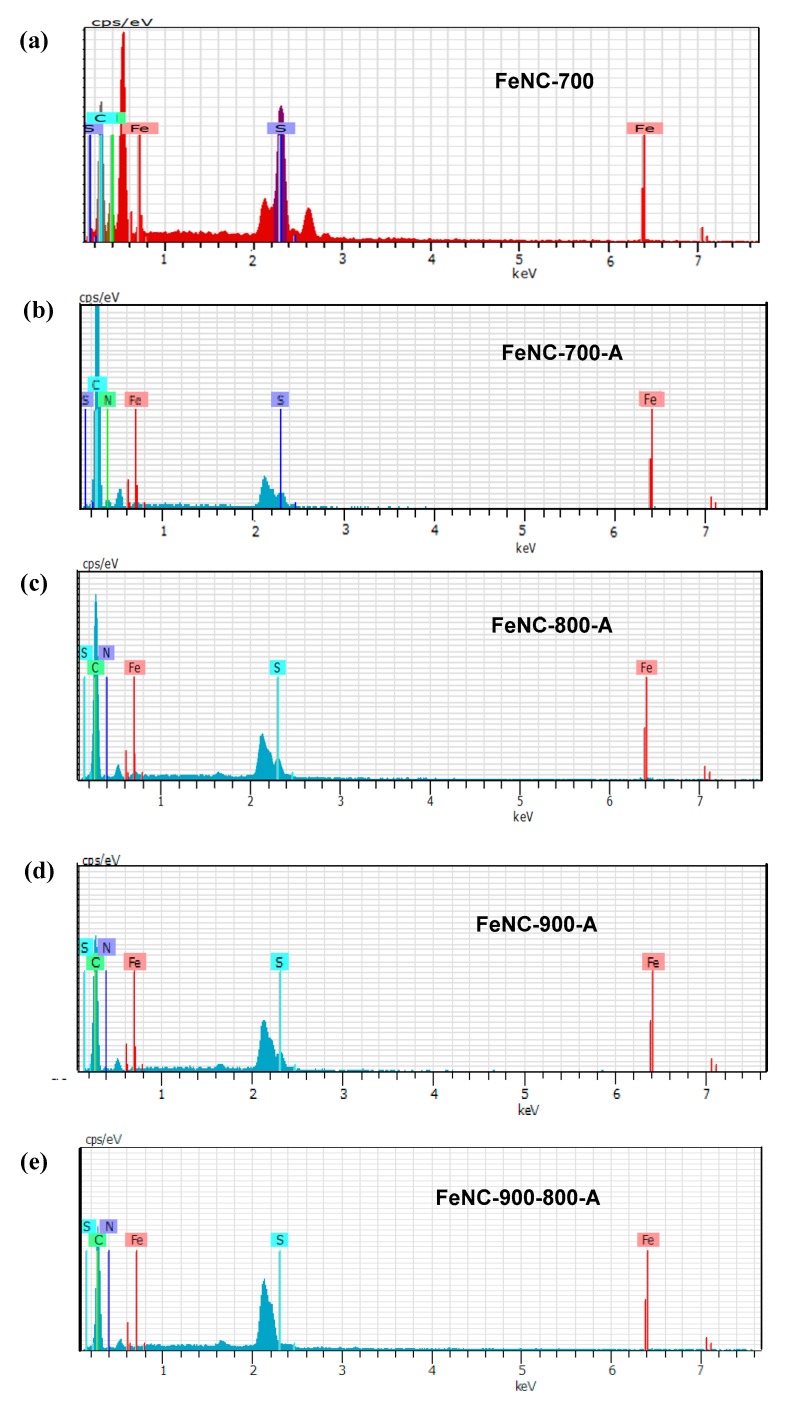
EDx spectra of various FeNxCs.

The atomic ratios of C, N, S, and Fe ( obtained from EDX spectra for all FeNxCs treated at various temperatures after acid-leaching ) are demonstrated in Figure 4 and listed in Table 1. The carbon composition increased with temperature in the first stage of calcination for FeNxCs and slightly decreased after second calcination (FeNxC-900-800-A) in an ammonia atmosphere, indicating NH_3_ did etch out some of the surface carbon and allowed for the formation of more Fe–N bonding [34].

Sample before acid-leaching demonstrates high S % in Figure 4a for FeNxC-700. And the low S % for all acid-treated samples reveal (Figure 4b–d) most of the crystallized FeS are removed. The absence of oxygen % for FeNxC-900-800-A indicates that magnetic Fe_3_O_4_ found before acid-treatment from X-ray diffraction has been removed by acid treatment (Figure 4e). N composition decreases with temperature and slightly increases after NH_3_ join in the second stage of calcination (Table 1). The Fe reaches the highest (3.84%) for FeNxC-900-800-A (Table 1), attributing to the formation of more Fe–N bonds in the presence of NH_3_, acting as both etching materials and N sources.

### 3.5. SEM

**Figure 5:**
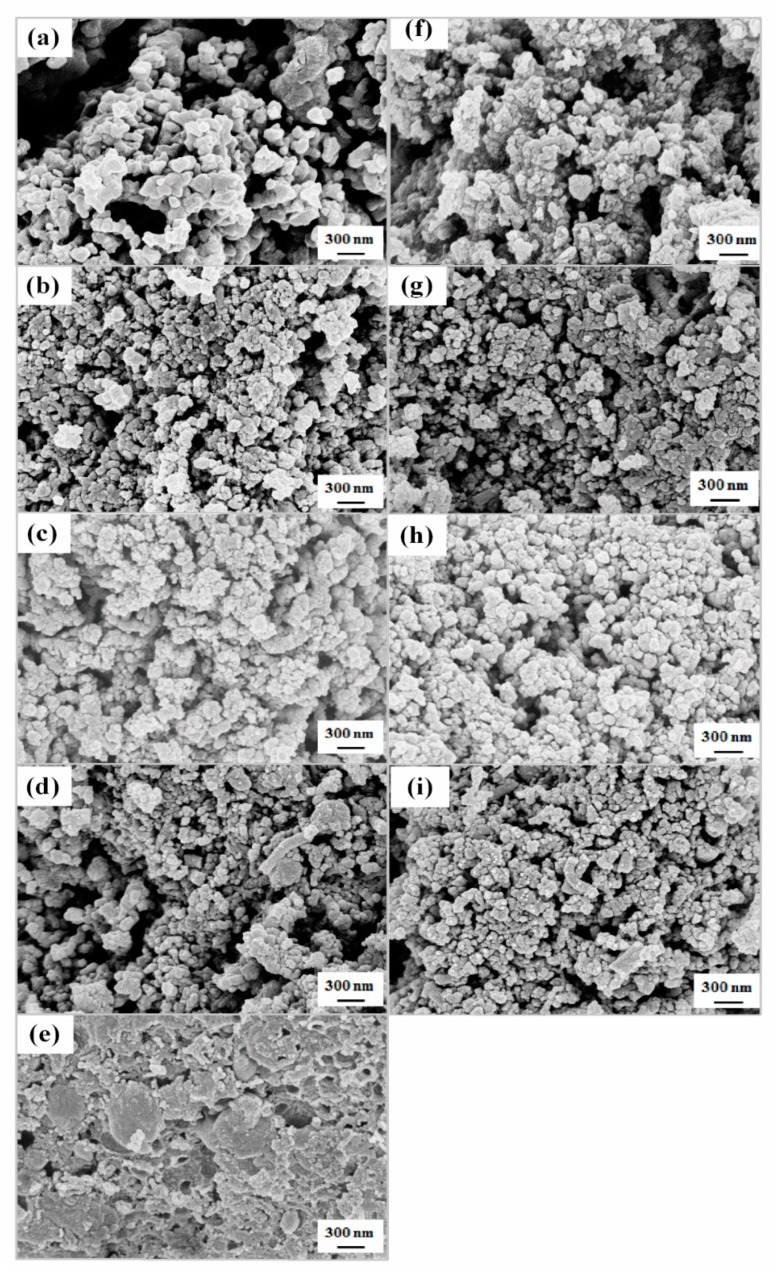
SEM micrographs of (**a**) neat PANI (**b**) FeCl_3_/EDA = 1/1 treated at 900 °C (**c**) 1/2 (**d**) 1/3 (**e**) 1/4 (**f**) FeNxCs treated at 700 °C (**g**) 800 °C (**h**) 950 °C (**i**) 900–800 °C.

The optimal ratio of FeCl_3_/EDA was also confirmed by taking the SEM micrographs at various ratios and illustrated in Figure 5a–e. Samples with ratios from 1/1 to 1/3 (Figure 5b–d) demonstrated micro- and mesopores on the surfaces after calcination at 900 °C. However, all the pores were covered with tiny Fe elements (Figure 5e) when the ratio was increased to 1/4, revealing the optimal ratio to be equal to the stoichiometric one (1/3).

The micrographs of all FeNxCs treated at different temperatures are illustrated in Figure 5f–i, which reveals the decreasing pore-size from large pores to mesopores with temperature for one-stage calcination. And the second calcination (two-stage) could have further shrunk the pore-size but still remained within the mesoporous range (Figure 5i). The surface area could have increased tremendously after second calcination in NH_3_ at 800 °C as well, which will be discussed in the following section.

### 3.6. BET Surface Area

**Figure 6:**
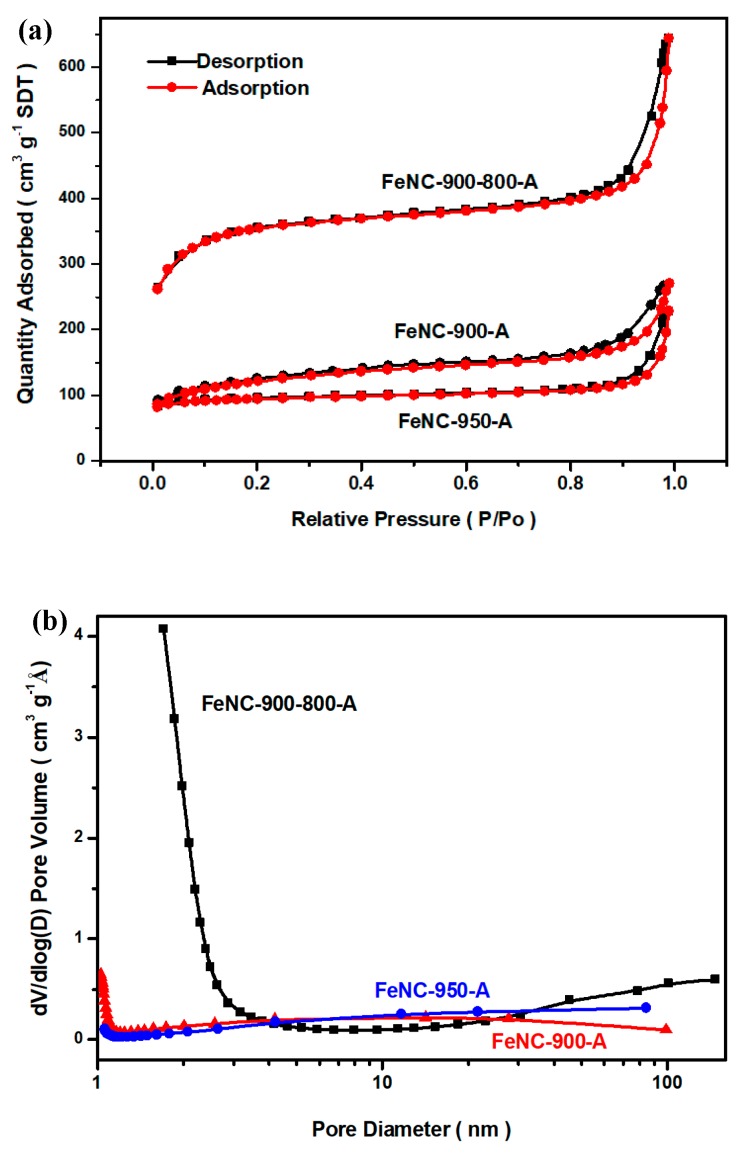
(**a**) N_2_ adsorption-desorption isotherms of acid-leaching FeNxCs (**b**) pore size distribution of acid-leaching FeNxCs.

The type IV isotherm, which is the characteristic N_2_-absorption and desorption curves of mesopores, can be clearly seen in Figure 6. FeNxC-900-800-A (Figure 6a) demonstrates much higher specific volume (>250 cm^3^·g^−1^) than either -900-A or -950-A (<200 cm^3^·g^−1^) at all relative pressures. Furthermore, the surface area (specific volume) became less and less with increasing calcination temperature during one-stage calcination in the N_2_ atmosphere. The collapsing destruction at a high temperature can result in the loss of more Fe and N elements and surface area, as already seen in Table 1 and Figure 6a, respectively. The specific area was significantly increased from 395 to 1098 m^2^·g^−1^ according to Table 2 and Figure 6a when FeNxC was calcinated again in an NH_3_ atmosphere at 800 °C.

Additionally, the pore size distribution illustrated in Figure 6b was obtained by Barrett-Joyner-Halenda (BJH) method, revealing the presence of both micro- and mesopores. The re-rising surface area found in Figure 6a and Table 2 for FeNxC-900-800-A could be attributed to the etching power of NH_3_ which caused only insignificant damages on the surfaces but created more micro- and mesopores in addition to compensating for loss of the N element. The graded distribution of pore sizes indicates FeNxCs are very useful to improve ORR because the presence of micropores can uncover the FeNxC active sites, and mesopores are able to play the roles of confining the O_2_ inside, effectively shortening the diffusion distances [39].

### 3.7. Oxygen Reduction Reaction (ORR) Performance

**Figure 7:**
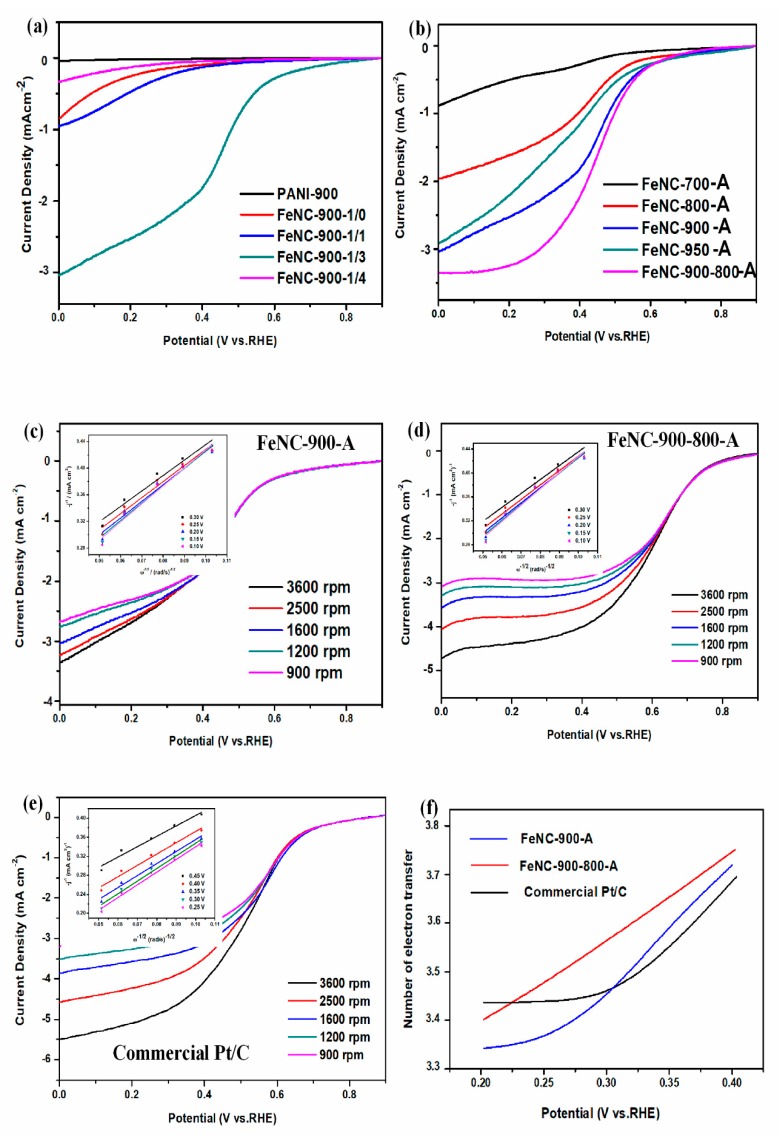
(**a**)–(**d**): LSV curves of various FeNxCs (**e**) LSV curves of commercial Pt/C (**f**) Numbers of e-transferred of various FeNxC compared to commercial Pt/C.

To evaluate the electrocatalytic activity of FeNxCs prepared with different FeCl_3_/EDA ratios and treated temperatures after acid-leaching, various LSV curves are measured and shown in Figure 7. After treated at 900 °C, neat PANI did not demonstrate any ORR current in Figure 7a though it is an N containing compound and calcination could also improve its conductivity. If ferric ions were introduced in the absence of EDA, the reduced current at zero potential increased to −0.95 mA·cm^−2^ in Figure 7a, indicating the electrocatalytic capability of iron transitional metal for ORR, depicted in Scheme 2, whose empty *d*-orbitals were able to accommodate (adsorb) oxygen for ORR. The presence of coordinated N is similar to the co-catalyst of Ziegler Natta, which behaves as the absorbing sites of unsaturated monomers owning double bonds like oxygen (Scheme 2). Likewise, with those Fe–Nx with certain empty coordinated sites, they are able to accommodate oxygen molecules with the right incoming angles that allow them to fit into the empty coordinated sites of iron centers and be captured by the FeNxC catalysts.

However, without the cooperation (complexation) of –Nx (in Fe–Nx form) from the EDA, the ORR can only occur in a lower scale. The joining of equal molar ratio of EDA (1/1) before calcination at 900 °C could slightly improve the reduced current to 1 mA·cm^−2^ in Figure 7a. A significant increase of reduced current to 3 mA·cm^−2^ when the molar ratio was elevated to 1/3 which is the stoichiometric ratio to form tri(ethylenediamine)iron(III) complex (Scheme 1). We believed the effective Fe–Nx bonding in the form of pyridinic, graphitic, or pyrrolic N were formed at this ratio by calcination at 900 °C (Scheme 1). The six coordinated Ns belonging to three EDAs chelated to Fe^3+^ will be reduced to less than five (Fe–N_2_, Fe–N_3_, or Fe–N_4_) after high temperature calcination and part of the Ns of EDA would join into the carbonization structure of PANI (Scheme 4).

Excess EDA did not further improve the ORR but almost deterred the ORR according to Figure 7a. The reduced current almost disappeared in its LSV curve due to the covered active sites (drastically decreased surface area, will be discussed in BET section). Therefore, the stoichiometric ratio 1/3 is the optimal ratio for obtaining the highest degree of ORR.

FeNxCs with FeCl_3_/EDA ratio of 1/3 were calcinated at various temperatures and the results were illustrated in Figure 7b. When the temperature was increased from 700 to 900 °C, it brought the improvement of reduced current according to Figure 7b. However, the current decreased when the temperature was over 950 °C due to the loss of Fe and N. The reduced current can be regained if it experiences second calcination in mixed gases of N_2_ and NH_3_. As expected, the reduced current was increased to 3.4 mA·cm^−2^ after second calcination (Figure 7b), which can create more Fe–Nx covalent-bondings and surface area as already discussed.

LSV curves were obtained from RDE at different rotating speeds to calculate the number of e-transferred from K-L equations and compared with commercial catalyst Pt/C ORR in Figure 7c–e. The LSV curves and K-L lines (insets) of FeNxC-900-A, FeNxC-900-800-A and commercial Pt/C were obtained and illustrated in Figure 7c–e, respectively. The numbers of e-transferred for each catalyst at different potentials are revealed in Figure 7f which shows the e-transferred numbers are all close to 3.7 but the two-stage calcinated FeNxC (900–800) own higher e-transferred number of 3.75.

### 3.8. Single Cell Testing

**Figure 8:**
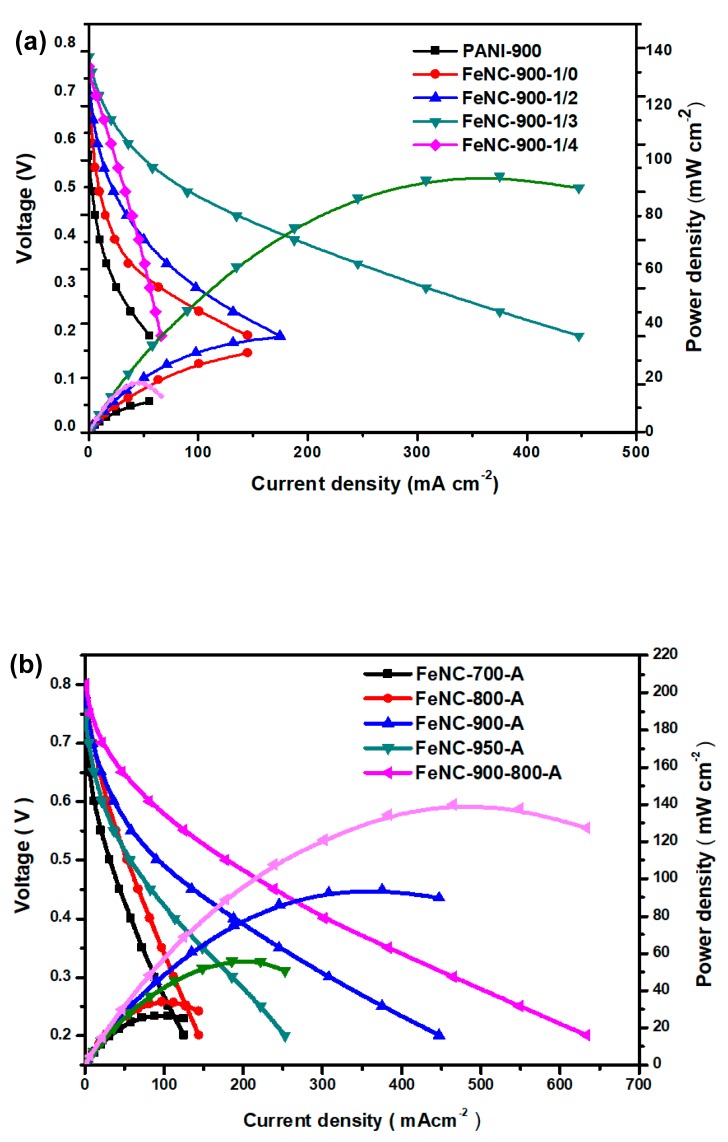
Power and current densities of single cells made of various FeNxC catalysts.

The single cell made of PANI-catalyst demonstrated no significant power or current density although it already owned carbonized after calcination according to Figure 8a. When only FeCl_3_ was enclosed by PANI in the absence of EDA, the obtained single cell demonstrated a slight increase in power and current densities, indicating the active Fe–N covalent bonding cannot be easily created with the covering PANI, although it is also an N source. The max. power (Pmax) and reduced current densities increased with increasing EDA from ratio 1/2 to 1/3 as seen in Figure 8a. The increase of these two types of densities revealed the importance of the complexation between FeCl_3_ and EDA which allow the Fe^3+^ to firmly capture the N of EDA through the coordinated bonding before calcination and converted into FeNxC covalently networks after calcination. However, when the ratio of FeCl_3_/EDA is over 1/3 (the stoichiometric ratio), additional EDA (1/4) did not bring the increase of either power or current density, but almost destroyed the entire power generation capability as shown in Figure 8a. We understand the excess EDA would reduce more tiny Fe elements which can seal all the micro- and mesopores as already seen in SEM micrographs in Figure 5e.

The Pmax can further increase to 140 mW·cm^−2^ when FeNxC (FeNC-900-800-A) went through a second stage of calcination in NH_3_ in accordance with Figure 8b, which also demonstrates that Pmax increased with treated temperatures at one-stage calcination, similar to the results obtained from LSVs (Figure 7b).

### 3.9. Stability Test

**Figure 9:**
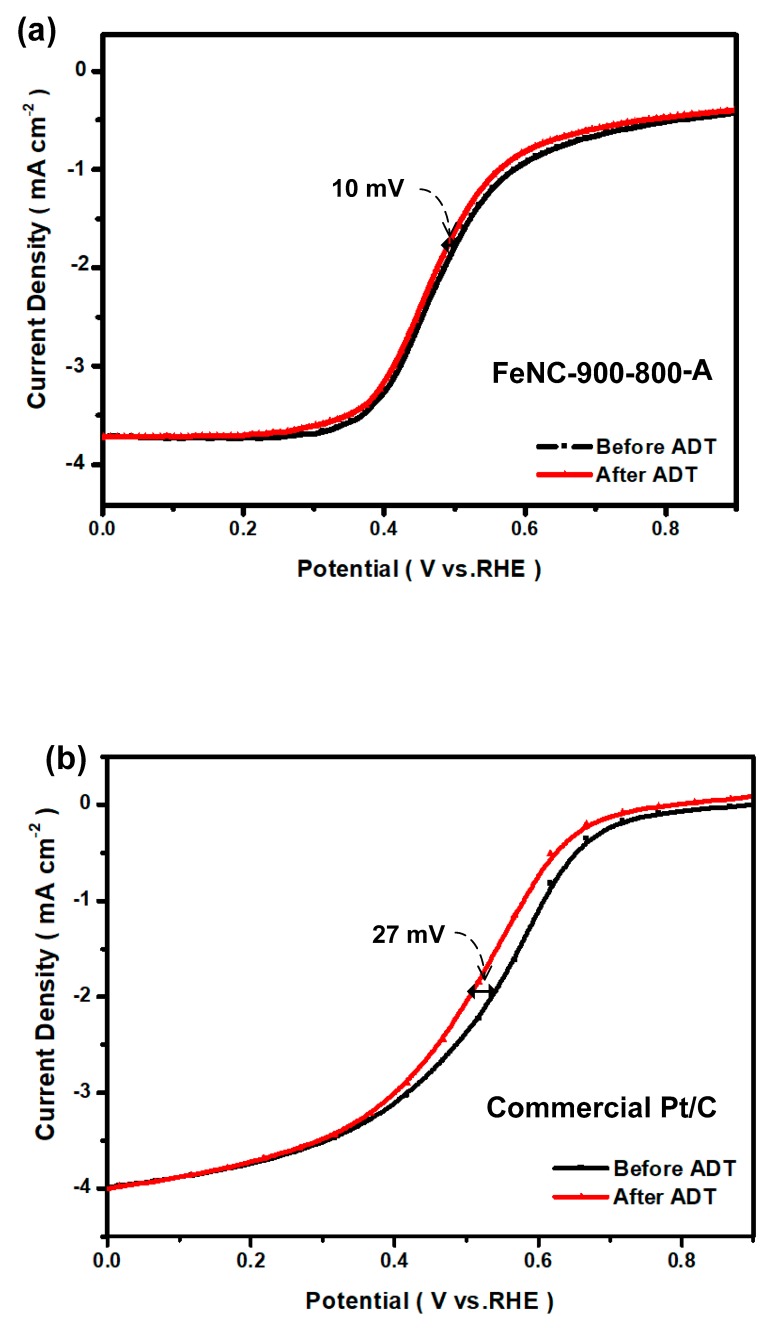
Accelerated durability test of (**a**) FeNC-900-800-A (**b**) commercial Pt/C catalyst.

A simple stability testing was performed by cycling the electrode between 0.0 and 0.9 V for 1000 cycles (about 3 h) in O_2_ saturated, 0.5 M H_2_SO_4_ (aq.) and forced the corrosion of the FeNxC catalyst, leading to the decrease of its reduced current. The half-wave-voltage (HWV) was measured after 1000 cycles at 1600 rpm for FeNC-900-800-A (Figure 9a) and compared with commercial Pt/C catalyst in Figure 9b. The HWV loss for FeNxC catalyst is only 10 mV compared to 27 mV for Pt/C, which proves the non-precious FeNxC catalyst is more resistant to the harsh acid electrolyte.

## 4. Conclusions

In order to create and enhance the covalent bonding between Fe and N, FeCl_3_ was complexed (neutralized) with EDA in the beginning, followed by the protection (covering) of PANI before thermal calcination to become an FeNxC catalyst.

FeCl_3_ was found to be able to complex with three EDAs (ligands) which occupied the six coordinated sites of the octahedral structure of Fe^3+^. The complexation brought the firm association of N with Fe^3+^, leading to the formation of Fe–N–C covalent bonding after calcination under the protection of PANI. The optimal ratio of FeCl_3_/EDA for better electrocatalytic performance of the resultant FeNxC catalyst is 1/3, as confirmed by IR-spectra, X-ray diffraction, LSV curves, SEM micrographs, and the higher power density of the obtained single cell.

Most of the FeNxC catalysts generated FeS crystals after calcination due to the persulfate initiator that applied to prepare PANI shell, which can be removed by acid-leaching according to X-ray diffraction. However, if FeNxC experiences second calcination in NH_3_, magnetic Fe_3_O_4_ is the dominant product that can also be removed by acid.

It was also found that the FeNxC, which experiences two-stage calcination (FeNC-900-800-A), one in N_2_ and the other in mixed gases of N_2_ and NH_3_, demonstrated the best ORR performance and obtained a higher power density due to the presence of high concentrations of both micro- and mesopores as characterized by SEM micrographs. The higher surface area of FeNC-900-800-A measured by BET method also contributed to the better electrocatalytic performance. The active sites of the FeNxCs were confirmed by XPS as the pyridinic, pyrrolic, and graphitic N centers, which can also accommodate the O_2_ and improve ORR.

The FeNxC based catalysts showed excellent performance and stability for ORR, which implies that it is a promising candidate as an electrocatalyst in PEMFC. In the future, we would try to substitute Fe with Co (Cobalt) and provides other types of amino-complexes with Co to further increase the electrocatalytic performance of the non-precious metal catalysts.

## Figures and Tables

**Table 1:** EDX elementary analysis (atom %) for FeNxC treated at various temperatures and after 1 M sulfuric acid-leaching.

Element	700 °C	800 °C	900 °C	950 °C	900-800 °C
C	81.35	84.00	84.54	88.07	87.00
N	12.35	9.01	9.11	6.91	7.38
S	4.02	3.83	3.65	2.30	1.78
Fe	2.27	3.06	2.91	2.72	3.84

**Table 2:** the specific surface area and averaged pore size of FeNxC.

Sample	BET surface area(m^2^/g)	Averaged pore diameter(nm)
FeNC-900	395	4.23
FeNC-900-800	1098	3.63
